# Development of the InCharge Health Mobile App to Improve Adherence to Hydroxyurea in Patients With Sickle Cell Disease: User-Centered Design Approach

**DOI:** 10.2196/14884

**Published:** 2020-05-08

**Authors:** Nicole M Alberts, Sherif M Badawy, Jason Hodges, Jeremie H Estepp, Chinonyelum Nwosu, Hamda Khan, Matthew P Smeltzer, Ramin Homayouni, Sarah Norell, Lisa Klesges, Jerlym S Porter, Jane S Hankins

**Affiliations:** 1 Department of Psychology St Jude Children’s Research Hospital Memphis, TN United States; 2 Department of Pediatrics Northwestern University Feinberg School of Medicine Chicago, IL United States; 3 Division of Hematology, Oncology and Stem Cell Transplant Ann & Robert H Lurie Children's Hospital of Chicago Chicago, IL United States; 4 Department of Hematology St Jude Children’s Research Hospital Memphis, TN United States; 5 Methodist Health Care Memphis, TN United States; 6 Division of Epidemiology, Biostatistics, and Environmental Health School of Public Health University of Memphis Memphis, TN United States; 7 Department of Foundational Medical Studies William Beaumont School of Medicine Oakland University Rochester, MI United States; 8 University of Illinois Health Institute for Healthcare Delivery Design Chicago, IL United States

**Keywords:** sickle cell anemia, hydroxyurea, hydroxycarbamide, medication adherence, self-management, mobile health, internet

## Abstract

**Background:**

Sickle cell disease (SCD) is an inherited blood disorder causing acute complications and chronic progressive end organ damage. SCD is associated with significant morbidity, early mortality, impaired health-related quality of life, and increased acute health care utilization. Hydroxyurea is a US Food and Drug Administration–approved medication that reduces disease complications, acute health care utilization, and costs. However, adherence to hydroxyurea is suboptimal. Mobile health (mHealth) interventions have the potential to improve hydroxyurea adherence, but few examples exist that are specific to the SCD population.

**Objective:**

This study aimed to design a mHealth intervention for individuals with SCD to improve adherence to hydroxyurea, using a user-centered design that was informed by specific barriers to hydroxyurea adherence and utilization in this population.

**Methods:**

This study consisted of 4 phases. In phase 1, individuals with SCD and health care providers participated in an optimization digital workshop. In phase 2, patients completed surveys pertaining to their interest in mHealth use, barriers and facilitators to hydroxyurea use, and health literacy. Phases 3 and 4 involved semistructured interviews and focus groups, respectively, and used the Health Belief Model (HBM) as the framework to investigate drivers of poor hydroxyurea adherence and to inform the development of an app prototype. In addition, in phase 4, we have incorporated the patients’ feedback on the preliminary app prototype and its features.

**Results:**

Barriers to hydroxyurea adherence were consistent with the literature and included forgetfulness and several specific thoughts and emotions associated with hydroxyurea use (eg, fear of side effects, depression, stigma, and hopelessness). In addition, more than half of the participants reported potentially low health literacy. Preferred patient app features included 7 key components, namely (1) medication reminders and tracker, (2) disease education, (3) communication, (4) personalization, (5) motivation, (6) support during pain episodes, and (7) social support. Utilizing a user-centered design approach, data obtained from patients and providers were translated into features within the app, mapping to components of the HBM and the specific drivers of hydroxyurea adherence and matching the literacy level of the population, resulting in the development of a novel mobile app called InCharge Health.

**Conclusions:**

The InCharge Health app is an mHealth intervention developed with substantial input from users and by mapping the HBM as the framework that guided the choice for its components. InCharge Health is a customized product for the SCD population aimed at optimizing medication adherence, with the end goal of improving quality of life and health outcomes among patients with SCD. The efficacy and implementation of the InCharge Health app as an mHealth intervention to promote hydroxyurea adherence will be tested in a future stepped-wedge multicenter trial for adolescents and adults with SCD.

## Introduction

### Background

Sickle cell disease (SCD) is a common inherited hemoglobin disorder in the United States, affecting approximately 100,000 Americans, mainly of African American descent [1-3]. SCD is a debilitating illness with several acute and chronic complications, including vaso-occlusive pain episodes, acute chest syndrome, cerebrovascular events, cognitive dysfunction, and progressive end organ damage. SCD has been associated with significant morbidity, early mortality, impaired health-related quality of life [3-5], and increased health care utilization [2,6]. Hydroxyurea (also known as hydroxycarbamide) is a medication approved by the US Food and Drug Administration (FDA) that has several health benefits and is cost-effective in pediatric and adult patients with SCD [7-19]. Hydroxyurea induces fetal hemoglobin production, thereby decreasing sickle hemoglobin erythrocyte polymers, hemolysis, and vaso-occlusion [10,11]. Rigorous investigation over the past 30 years has demonstrated the efficacy of hydroxyurea in reducing disease complications, health care utilization, and costs for patients with SCD [7-18]. Consequently, the National Heart Lung and Blood Institute (NHLBI) has issued guidelines recommending its use among symptomatic adults and all children with SCD (HbSS and HbSβ^0^-thal genotypes) aged ≥9 months [19]. Despite being evidence-based and endorsed by NHLBI guidelines, adherence to hydroxyurea in SCD remains suboptimal [7,20-23]. Lower adherence rates have been associated with worse health outcomes, including more frequent SCD-related complications, low health-related quality of life, and increased health care utilization [4,7,15,24,25].

Medication adherence is a dynamic multifactorial process that reflects influential factors at three different levels: patient, health care provider, and health system [26]. In SCD, at the patient level, various barriers related to hydroxyurea adherence have been identified, such as concerns about efficacy and side effects, forgetfulness, inability to obtain refills, and lack of knowledge about hydroxyurea [20-23,25,27-33]. In addition, among patients with SCD, the number of adherence barriers is negatively correlated with the overall adherence rates to different medications, including hydroxyurea [20]. Optimizing hydroxyurea adherence throughout the lifespan is critical to improving health outcomes in this population, particularly among adolescents and young adults—a vulnerable subpopulation at a higher risk of low hydroxyurea adherence and disease-related morbidity and mortality [1-3,19,24,34,35]. In addition, adolescents and young adults are likely to start or are already managing their medications independently, making this an ideal developmental period to engage patients with SCD in building their self-management skills, including adherence to hydroxyurea, which is essential and could have long-term benefits [36-38]. Furthermore, achieving a better understanding of different patient barriers represents a key strategy for improving hydroxyurea adherence in SCD [20,23,25,31,33].

The Health Belief Model (HBM) is commonly used to explain the constructs underlying medication adherence behavior [39]. The HBM suggests that patients’ adherence level is related to a number of factors that are independently weighed [39]. The health-related action driving the increased use of hydroxyurea includes 5 constructs within the HBM: perceived susceptibility, perceived severity, perceived benefits, perceived barriers, and self-efficacy. Notably, these 5 constructs represent modifiable factors that, together, can be influenced to increase the use of hydroxyurea.

Technological solutions are becoming more common in health care. Access to personal technology is nearly ubiquitous, and a growing number of patients use technology for their health care needs [40,41]. Mobile health (mHealth) interventions have been shown to improve patient activation and engagement [42-46], making them a possible tool to improve outcomes. In particular, earlier studies showed that individuals with SCD and their families were interested in using mHealth technologies to manage their health [22,47,48]. In other chronic health conditions (eg, asthma and diabetes mellitus), there is mounting evidence that self-care and self-management skills can be improved with the use of mHealth interventions [49-51]. Furthermore, recent systematic reviews showed promising data to support the overall feasibility, acceptability, and efficacy of mHealth interventions in promoting adherence behavior and improving health outcomes in different patient populations [52-57], including SCD [58]. In this study, we examined barriers and facilitators to hydroxyurea adherence informed by the HBM to support the development of a new mHealth intervention to foster greater hydroxyurea use.

### Study Objectives

To date, only a few reported mHealth interventions have focused on medication adherence among individuals with SCD [58-61]. Additionally, the integration and application of user-centered design principles in the development of interventions have been limited within this population [58,62]. User-centered design has been defined as “an iterative design process in which designers focus on the users and their needs in each phase of the design process, with a call for involving users throughout the design process via a variety of research and design techniques so as to create highly usable and accessible products for them” [63]. The objectives of this study were to (1) use an agnostic approach to identify patient-level barriers to hydroxyurea adherence and utilization and (2) apply a user-centered design approach to develop a patient-informed SCD mobile app, *InCharge Health*, as a behavioral intervention to improve adherence to hydroxyurea. We hypothesized that hydroxyurea adherence barriers would vary among patients with SCD and that the barriers could be targeted by functionalities within an mHealth app.

## Methods

### Design

This study utilized user-centered design, which is an evidence-based and iterative approach that incorporates the needs and context of a specific end-user group (eg, adolescents and young adults with SCD) and helps ensure that the resulting digital health intervention is acceptable and effective [62,64]. The first step of this approach is to better understand the users’ needs, context, and experiences [62,64]. Thus, phases 1 to 3 of this study utilized a mixed method approach, with qualitative data derived from an optimization digital innovation workshop (phase 1) and semistructured interviews (phase 3), and quantitative data derived from a needs assessment survey (phase 2). Together, these data were used to better understand the needs and experiences of individuals with SCD who aim to consistently take hydroxyurea and to inform the initial design of an app prototype. Consistent with the iterative nature of user-centered design, phase 4 of the study involved obtaining end-user feedback on the app prototype via focus groups to further tailor and inform the final design of the app.

### Participants

Individuals were eligible if they were between the ages of 15.0 and 44.9 years, had a diagnosis of SCD, and were affiliated with one of the multicenter National Institutes of Health (NIH)–funded SCD implementation consortium (SCDIC) sites. The development of *InCharge Health* is part of the multicenter NIH-funded SCDIC that aims at increasing the adoption of evidence-based interventions, including hydroxyurea treatment, among individuals aged 15.0 to 44.9 years with SCD using implementation science approaches [65].

Prior use of hydroxyurea was not an inclusion criterion for the study. Participants with an indication of hydroxyurea therapy, but no prior history of receiving this therapy, or who received this therapy but later discontinued it, were included as we also sought to obtain the perspectives of individuals who had previously been offered hydroxyurea but declined to take it, as such perspectives could provide further insight into potential barriers to taking hydroxyurea. Participants were recruited continuously from 2 SCDIC hematology clinics (St Jude Children’s Research Hospital and Methodist University Hospital) and a community-based organization in Memphis, Tennessee, United States. Participants were recruited from these specific clinics as these were SCDIC sites. Recruitment took place during regular clinic visits. Participants received information about the study either via a flyer or via in-person communication with a study team member. All participants signed an informed consent before any study procedure. If participants were minors, their legal guardian signed the consent, and assent was obtained from the adolescent participants. The study was approved by the Institutional Review Boards from St Jude Children’s Research Hospital and the Methodist Healthcare/University of Tennessee Health Science Center.

### Procedure

This study addressed the patient underutilization of hydroxyurea by developing a new mHealth intervention to improve hydroxyurea adherence. The study consisted of 4 phases, with a total of 118 participants. *Phase 1*: patients (n=6) and health care providers (n=12) participated in an optimization digital innovation workshop [62]. *Phase 2*: patients (n=99) completed surveys pertaining to demographic information, mHealth use, perceived barriers and facilitators to hydroxyurea use, and health literacy. *Phase 3*: patients (n=20) completed semistructured interviews pertaining to their experiences with apps and mobile phones, perceptions of hydroxyurea, perceptions of risk of SCD complications, and their SCD self-efficacy. *Phase 4*: patients (n=12) participated in focus groups to provide feedback on an initial app prototype. Feedback on the app and several of its proposed features were used to further refine the app prototype. Patients were compensated for their time in each phase of the study as follows: *Phase 1*, US $100; *Phase 2,* US $15; *Phase 3*, US $15; *Phase 4*, US $20. Completion of all study phases took 1 year in total.

#### Phase 1: Optimization Digital Innovation Workshop

Patients and providers were invited to participate in a 1-day workshop focused on identifying problems around hydroxyurea use and developing potential solutions. This workshop lasted 8 hours and included patients, clinicians, both hematologists and psychologists, researchers, and research staff. Preferences for features of the app were also identified through an iterative process and group discussions that started with the identification of gaps in hydroxyurea utilization (specifically why adherence was low) and ended with possible digital solutions to foster greater hydroxyurea use.

#### Phase 2: Needs Assessment Survey

This survey consisted of a total of 20 questions that aimed at investigating the barriers and facilitators to hydroxyurea use, patients’ perception of the mHealth benefit, and the health literacy level of the population. The 4 main sections of the survey were as follows:

- Demographics: patients completed a demographic survey that assessed their age, gender, race, ethnicity, SCD type, zip code, household income, and household size.
- mHealth use: patients completed a 2-item study-specific survey assessing whether they are currently using or ever used mHealth to help them take their hydroxyurea.
- Hydroxyurea use and barriers: patients completed a 7-item study-specific survey assessing hydroxyurea use (eg, current and past use, if any, and duration if currently using hydroxyurea) and perceived barriers to hydroxyurea use.
- Health literacy: patients completed the Newest Vital Sign (NVS) [66], a 6-item validated screening tool that assesses an individual’s ability to understand and apply information displayed on a nutrition label for ice cream. This tool measures both health literacy and numeracy.

#### Phase 3: Semistructured Interviews

**Figure 1:**
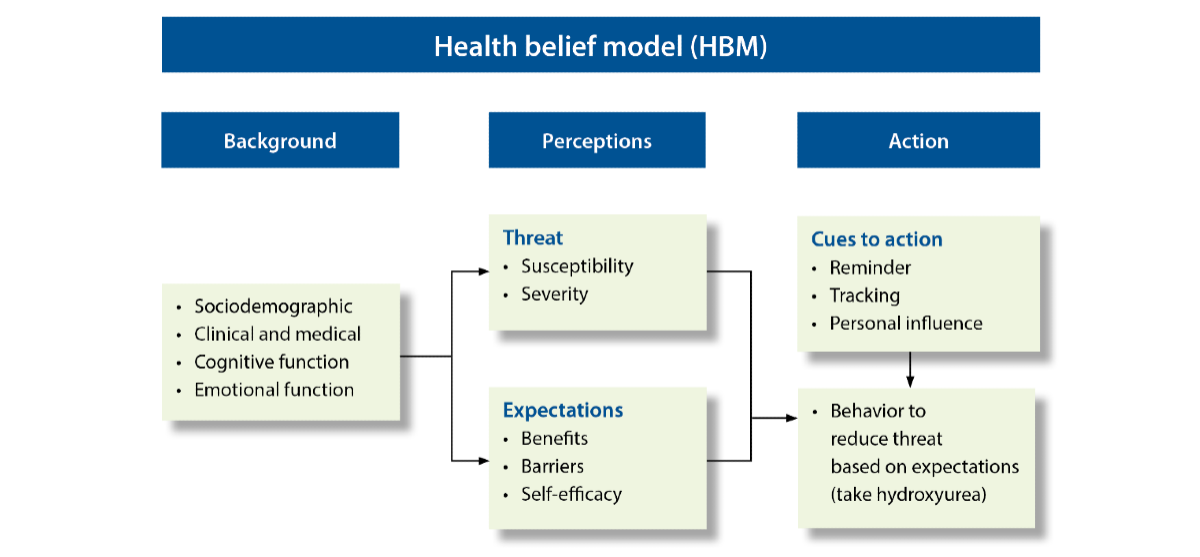
Health belief model applied to hydroxyurea adherence.

Individuals who agreed to participate in the survey were invited to take part in a 30 to 45-min semistructured interview that provided complementary data on the participant’s knowledge and perceptions of hydroxyurea. Interview participants were also asked about their prior history with mHealth apps for medication adherence and perceptions. Interviews were conducted in the sickle cell clinic by a single study team member. The HBM [39] was used as a theoretical framework for the development of the final interview guide. Figure 1 illustrates the HBM as applied to adherence to hydroxyurea. Participants were asked specific questions relevant to the model, including questions about perceived barriers and benefits to taking hydroxyurea, perceived level of risk of having SCD complications, and self-efficacy regarding their ability to take care of their health.

#### Phase 4: Focus Groups

Individuals who completed the surveys were asked to indicate their interest in participating in a focus group in the future. Those indicating an interest, and who had not already participated in the semistructured interviews, were invited to participate in the focus groups. The overall goal of the focus groups was to obtain feedback on the app prototype to assist with further app refinement.

### Data Synthesis and Analysis

Quantitative data were analyzed using SPSS version 25 (IBM, Armonk, New York). Descriptive and correlation analyses were used to summarize demographic and survey item data. With regard to the qualitative data obtained in phase 3 and phase 4*,* interviews and focus group discussions were audiotaped, transcribed, and entered into NVivo 12 for coding and analysis. Coding of the interview data was conducted by two members of the study team (JH and HK) following a cyclical coding process using a constructivist grounded theory approach [67-69]. A final coding scheme was developed using a consensus coding approach where study team members met weekly to discuss agreement and disagreement and rectified any discrepancies throughout the coding process [68]. The cyclical coding process involved 3 coding steps—Step 1: an initial set of codes on the basis of interview guide questions; Step 2: a subset of codes within Step 1 codes; Step 3: thematic codes identified by common codes found within Step 1 and 2 codes.

## Results

### Study Population

In phase 1**,** participants were 6 adult patients with SCD between the ages of 18 and 45 years (3 males and 3 females) and 13 health care providers, community advocates, and researchers, including 2 physicians, 1 nurse, 2 psychologists, 4 PhD researchers (behavioral and informatics), 1 health information security expert, 1 community-based organization member, and 2 research coordinators; 10 patients were not interested in participating in phase 1. Purposive sampling was used to recruit patient and provider/researcher participants to phase 1 of the study. Table 1 presents demographic data for patient participants in phases 2, 3, and 4. In phase 2, 100 participants were surveyed, and 99 patients completed the demographic survey, who were separated by age group in Multimedia Appendix 1. The upcoming clinic visits of potential patient participants were reviewed, and patients were randomly approached during their visits to participate in phase 2 of the study. Participant demographics were comparable to the larger SCD population [70]. In phase 2, 10 participants declined to participate; 12 declined to participate in phase 3, and 19 declined to participate in phase 4. The mean age of patients was 21.7 years (SD 6.4) and 46% (46/99) were female. Adolescents (aged 15-17.9 years) comprised 78% (78/99) of the total participants, and 21% (21/99) were between the ages of 18 and 44.9 years. Overall, 70% (70/99) of patients had HbSS or Sβ^0^ thalassemia genotypes, and the remaining had HbSC, HbSβ^+^ thalassemia, or another variant. Almost all patients were African American (94/99, 94%), with the remaining 5.1% indicating “Other” and 1% choosing Hispanic or Latino as their ethnicity. For household income, 38% of participants either did not know or preferred not to answer, 47% had a household income of US $29,999 or lower, 9% indicated a household income between US $30,000 and US $59,999, and 6% were above US $60,000. These patients were randomly selected during their clinic visits, and their demographics were similar to the demographics of the longitudinal cohort study Sickle Cell Clinical Research and Intervention Program (SCCRIP), which encompasses nearly the entire population of the participating sites [70].

During clinic visits, participants who completed the survey were offered the opportunity to participate in the interviews until a sample size of 20 was reached. Thus, 20 adolescents and adults (average age 24.5 years, SD 9.28; 45% female) participated in *phase 3* of the study, which involved semistructured interviews. In *phase 4*, 12 adolescents and adults (average age 21.10 years, SD 6.01; 75% female) participated in focus groups where they were invited to review an interactive app prototype and provided feedback on functionality, key features (eg, medication reminders, tracking, and points/rewards), and overall design.

**Table 1:** Participant characteristics across phases 2, 3, and 4.

Characteristics		Phase 2 enhanced NA^a^ survey, (N=99)	Phase 3 interview, (N=20)	Phase 4 focus group, (N=12)^b^
Age (years), mean (SD)		22 (6.46)	24 (9.28)	21 (6.01)
**Age group (years), n (%)**				
	15-17	31 (31)	7 (35)	5 (42)
	18-24	47 (48)	7 (35)	5 (42)
	25-34	9 (9)	1 (5)	1 (8)
	35-45	12 (12)	5 (25)	1 (8)
**Gender, n (%)**				
	Female	46 (46)	9 (45)	9 (75)
	Male	53 (53)	11 (55)	3 (25)
**Race n (%)**				
	African American	71 (96)	19 (95)	12 (100)
	Other	3 (4)	1 (5)	—^c^
**SCD^d^** **genotype, n (%)**				
	HbSS	62 (63)	12 (60)	8 (67)
	HbSβ^0^ thalassemia	8 (8)	1 (5)	—
	HbSC	23 (23)	5 (25)	3 (25)
	HbSβ^+^ thalassemia	4 (4)	1 (5)	—
	Other variant	1 (1)	—	1 (8)
	Do not know	1 (1)	1 (5)	—
**Annual household income (US $), n (%)**				
	Less than 5000	23 (23)	2 (10)	2 (17)
	5000-9999	6 (6)	2 (10)	1 (8)
	10,000-14,999	6 (6)	2 (10)	—
	15,000-19,999	2 (2)	1 (5)	—
	20,000-29,999	9 (9)	—	1 (8)
	30,000-39,999	—	—	—
	40,000-49,999	6 (6)	1 (5)	1 (8)
	50,000-59,999	3 (3)	1 (5)	—
	60,000-79,999	3 (3)	—	2 (17)
	80,000-94,999	2 (2)	—	—
	95,000 and above	1 (1)	—	—
	Prefer not to answer	13 (13)	1 (5)	3 (25)
	Do not know	25 (25)	10 (50)	2 (17)
**NVS^e^** **health literacy, n (%)**				
	High likelihood of limited literacy	57 (58)	11 (55)	7 (58)
	Possibility of limited literacy	21 (21)	6 (30)	2 (17)
	Adequate literacy	21 (21)	3 (15)	3 (25)

^a^NA: needs assessment.

^b^A proportion of patients participated in >1 study phase.

^c^No participants in this category.

^d^SCD: sickle cell disease.

^e^NVS: Newest Vital Sign.

### Phase 1

During the optimization digital innovation workshop (Multimedia Appendix 2), patients and providers discussed obstacles to taking hydroxyurea, which were then categorized and prioritized. Identified obstacles included (1) side effects, (2) quantity and size of the pills, (3) busy life and a tendency to forget medications, (4) cost of the medication, and (5) attitudes toward medication and negative emotions (eg, depression, “tired of taking medication,” “no immediate or visible reward,” stigma, anger, and hopelessness). Group discussion also focused on features to include in the app, which derived 7 categories: (1) gaming, competition, and rewards; (2) group support and community; (3) education, resources, and ability to contact a doctor/an expert; (4) reminders and notifications; (5) personalization; (6) visual checklists; and (7) graphical trackers and charts. Participants also reflected on the most important features of the app. Overall, features that were considered the most important included reminders to take hydroxyurea, followed by personalization and setting individual goals/motivations, motivation messages, gaming and points, tracking graphs, and finally features that allow communication with friends/family if the user forgets to take hydroxyurea.

### Phase 2

#### Mobile Health Use

Overall, 24% (24/99) of those with a history of taking hydroxyurea had prior experience with text messages as an aid in taking hydroxyurea; 11% (11/99) indicated that they were currently using text messages to help them take hydroxyurea.

#### Hydroxyurea Use and Barriers

Most patients (72/99, 73%) were taking hydroxyurea at the time of survey completion (current users), and 6% had taken hydroxyurea at some point in the past (previous users). Among those currently taking hydroxyurea, 43% (43/99) had taken it for >6 years, 42% (42/99) had taken it between 1 and 5 years, and 9% (9/99) had been on hydroxyurea for <1 year. Among patients who had any history with hydroxyurea, 79% (78/99) indicated “forgetting to take the medicine” and 24% (24/99) reported “it was hard to take the medicine at the right time” as the leading barriers to hydroxyurea adherence. The next most frequently endorsed barriers included “I don’t like to think about having sickle cell disease when I am feeling well” (11/99, 11%) and “I am worried about side effects” (10/99, 10%). Of the total patients with a history of hydroxyurea use, 33% (33/99) indicated experiencing no barrier to hydroxyurea use. Facilitators to hydroxyurea use were rarely cited by the participants, who rather reported facilitators in the context of mHealth solutions.

#### Health Literacy

Most patients were found to have either a high likelihood of limited health literacy (57/99, 58%) or possibly limited health literacy (21/99, 21%). Only 21% (21/99) of patients were found to have an adequate level of health literacy.

### Phase 3

Results from phase 3 are presented around the primary content areas explored during the semistructured interviews and in alignment with the core modifiable components of the HBM as applied to this study: background, perceptions pertaining to expectations, perceptions pertaining to threat, and cues to action. 

#### App and Mobile Phone Experiences and Perceptions

All participants indicated regular use of mobile phone apps for social media, communication with peers or family, and entertainment consumption (eg, to watch movies, play games, listen to music, or read books). A minority of participants stated experience with using an app to assist with health-related activities, such as using the phone’s reminder function for medical appointments and medications or tracking physical activity. Overall, participants expressed positive perceptions about mobile phones and apps; however, when asked to state any concerns they might have, ensuring the protection of private data and general negativity in the realm of social media were the most common drawbacks to mobile phone apps.

#### Health Belief Model Perceptions: Benefits, Barriers, and Sickle Cell Disease Self-Efficacy

##### Education

Overall, most participants did not perceive a need for additional education on hydroxyurea, although some did indicate additional education could be helpful, including information on the pros and cons of taking hydroxyurea.

##### Perceived Benefits of Taking Hydroxyurea

The primary perceived benefit of taking hydroxyurea was the improvement of overall health and quality of life. Participants specifically mentioned the decrease in pain crises and a decrease in hospitalizations as benefits of hydroxyurea.

##### Barriers to Taking Hydroxyurea

A large majority of the participants identified forgetting to take hydroxyurea as a primary barrier to adherence. Additional barriers included insurance or price, number of pills in the dose, fatigue, competing activities (eg, busy schedule), and feeling “tired” of taking medications. Participants who were not currently taking hydroxyurea described several barriers to beginning or restarting hydroxyurea including hearing about someone who experienced side effects, currently taking several medications, never having been offered hydroxyurea by a physician, personal preference, and a lack of need to take it because of perceived low SCD severity.

##### High Sickle Cell Disease Self-Efficacy

Overall, the vast majority of participants described feeling confident or very confident about their ability to take care of their health, attend their medical appointments, and take hydroxyurea. Most participants also rated this confidence as 8 or 10 out of 10 (10 being the most confident).

#### Health Belief Model Perceptions: Susceptibility to Sickle Cell Disease Complications, Perceived Severity, and Sickle Cell Disease–Related Worry

##### Low Perceived Risk

Overall, most participants viewed themselves as at low risk of SCD complications. When asked why they are at low risk, participants reported engaging in strategies such as taking their medications, drinking water, dressing for the weather, and abstaining from risky health behaviors (eg, drinking alcohol and smoking). For those who saw themselves as being at high risk, reasons for this perception included experiencing elevated pain and an increased number of surgeries.

##### Increased Worry About Complications

Interestingly, although most participants perceived themselves as being at low risk for SCD complications, most also reported experiencing worry about SCD complications, maintaining good health, and negative health-related outcomes such as hospitalization, blood clots or stroke, and the cumulative damage of SCD. The occurrence of pain and pain crises was the most common worry reported by participants. It should also be noted that a subset of participants denied experiencing worry about SCD complications.

#### Health Belief Model Cues to Action: Preferred Features in an App for Hydroxyurea Adherence

##### Trackers and Reminders

Several participants indicated that they would like to have reminders within the app, including the ability to customize these reminders on the basis of their personal preferences. Participants also indicated it would be helpful to have the ability to track their adherence to hydroxyurea and other medications.

##### Education and Communication

Some participants also expressed a desire for the app to provide additional information about hydroxyurea (eg, dosage), and some expressed a desire to connect with other patients and friends and family (eg, parents) to increase accountability for taking hydroxyurea. Some participants also described a desire to communicate with their medical team via the app.

##### Engaging and User-Friendly

Overall, there was a general desire to have an app that was easy to use while also engaging. In being able to customize reminders, several participants reported that they would like to have the ability to make unique and exciting medication reminders linked to their personal motivation for staying healthy.

### Phase 4

**Figure 2:**
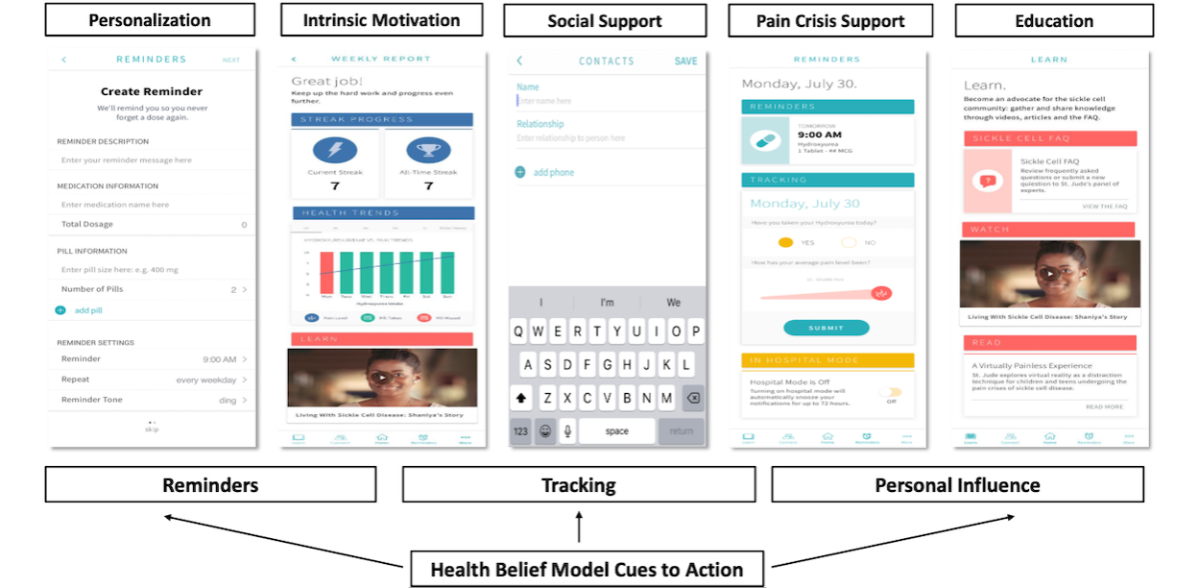
Screenshots of different functions in InCharge Health App mapped to health belief model.

Data derived from the focus groups revealed 5 primary themes: (1) personalization, (2) intrinsic motivation, (3) social support, (4) support during pain crisis, and (5) education. Participants’ input from this phase led to the development of the *InCharge Health* app (Figure 2).

#### Personalization

Participants expressed a desire to personalize various aspects of the app, including reminders, motivational messages, and educational content. Among adolescents, in particular, being able to put reminders in their own words was viewed as making the app feel less like “nagging.” Overall, participants also indicated that they would be less likely to ignore reminders if they were in their own words. As a result of this feedback, within the *InCharge Health* app, patients have the option of customizing the reminder description and the day and time that they receive the reminder. The customized reminder feature is shown in Figure 2.

#### Intrinsic Motivation

Contrasting views with regard to motivation to use the app were found among adolescents and adults. Specifically, adolescents liked the idea of short-term rewards being offered to increase motivation, whereas adults generally held the view that motivation should be more intrinsically based and come from a personal desire to support health and well-being. To accommodate both views, the concept of *streaks* (see Figure 2) was utilized within the app, such that participants can earn streaks when they take their hydroxyurea several days in a row and can, therefore, track their progress. In addition, participants can rate their pain each day and are provided with a weekly tracking report of their pain, which is, in turn, graphed next to their adherence to hydroxyurea (Figure 2). The rationale behind this feature is that directly showing patients changes in pain over time and how these are connected to changes in adherence to hydroxyurea will potentially increase patient motivation to take hydroxyurea consistently.

#### Social Support

Participants also expressed a desire to connect with others with shared experiences. Many reported already connecting with others with SCD via social media and online forums and liked the ability to connect with peers and experts all in one place. In response to this feedback, on the homepage of the *InCharge* *Health* app, patients have the option of connecting with their doctor, their medical chart, and other patients via the website, *one SCDvoice* [71]. They also have the option of adding *accountability contacts* who are sent messages when the patient does not indicate taking their medication for >4 hours (Figure 2).

#### Pain Crisis Support

Participants also expressed an interest in app features that would help provide support during a pain crisis. In response to this feedback, patients can monitor their pain across time by recording their daily pain level within the app (Figure 2). In addition, if a pain crisis occurs, patients can contact their clinic directly from within the app, access educational information about pain treatment, and talk to other patients about their experiences with pain.

#### Education

Participants also expressed interest in having access to educational content—both for themselves and for friends and family members. Specifically, participants were interested in content related to how hydroxyurea works, side effects, and pain management and tracking. In response, the *InCharge Health* app includes educational content pertaining to SCD, hydroxyurea, and more general health topics (eg, depression; Figure 2). Moreover, given the low levels of health literacy observed in phase 2, education and information more generally were provided in the app via several visual prompts (eg, graphs and images) and multiple formats, including videos, written materials (PDF files), and links to other websites. Additional app features accounting for low levels of health literacy observed included easy-to-read and simple instructions and links to the electronic medical record and the clinic to assist with navigation of the health system.

The HBM components of perceived barriers, cues to action, and self-efficacy emerged in the survey, semistructured interview, and focus group results and were subsequently incorporated in the development of the *InCharge Health* app (Figure 2). Forgetting to take medication was the largest reported barrier to hydroxyurea adherence. The app addresses this with cues to action with text messaging reminders that can be personalized by the user, and the user can enter accountability contacts who are notified if the user does not take their medication within the specified time period as indicated through the app. Participants also reported concern about medication side effects as a barrier; therefore, the app provides educational content through readings and videos. Additionally, the app tracks consistent hydroxyurea use through *streaks*, and users can track their pain experience and see how pain and medication use are connected, which in turn can increase intrinsic motivation and self-efficacy.

## Discussion

### Principal Findings

Hydroxyurea is one of only 2 FDA-approved medications for SCD, and the one with the most amount of evidence for its efficacy. Despite its evidence for clinical benefit, patients with SCD face difficulty in maintaining adequate adherence. Using a user-centered approach that investigated the reasons for poor adherence on the basis of the HBM, we identified the important drivers of poor adherence and translated them into an mHealth solution that addresses all important barriers, the *InCharge Health* app.

The results of this study indicated that there is an inadequate level of health literacy among adolescents and young adults with SCD, which is consistent with prior research showing that health literacy is suboptimal among adolescents [72,73] and adults with SCD as well as caregivers [73]. Prior research in other chronic diseases has also shown low health literacy to be related to poor medication adherence [74], thus further highlighting the need to provide appropriate and tailored education to patients with SCD who are attempting to consistently take hydroxyurea. Importantly, the *InCharge Health* app was developed accounting for the limited health literacy level of our population. Specifically, text information within the app was purposely written in a layperson format and at a sixth-grade reading level to increase the patients’ ability to understand the information provided. In addition, education and information more generally were provided via several visual prompts and multiple formats. In sum, these multi-component features are simple, intuitive, and aim to provide easy-to-understand information to patients on their condition and effective treatments. Furthermore, as low health literacy is likely strongly associated with both education level [72] and cognitive functioning among individuals with SCD, our findings have implications for future investigations examining the influence of socioeconomic factors and executive functioning among youths and adults with SCD. Within the SCCRIP study [70], we examined the relationship between health literacy and executive functioning and educational level. Results obtained from these studies will assist in informing future interventions targeting health literacy.

### Barriers to Hydroxyurea Adherence

SCD patients likely engage in an implicit assessment or an individual cost-benefit evaluation where possible benefits from hydroxyurea are weighed against concerns about using it [75-77]. Our findings showed that a significant number of our adolescents and adults with SCD had different positive and negative views about hydroxyurea, which could affect their perceptions of the necessity of using it, which was consistent with earlier reports in SCD [20,23,27,55,78] and other chronic medical conditions [75,77,79-86]. In a study by Haywood et al [27], SCD patients reported a number of concerns related to the use of hydroxyurea, including insufficient knowledge, lack of perceived benefits, and possible side effects, similar to our findings. In support of our findings, forgetfulness has been a common reason for low medication adherence among individuals with SCD and other patient populations [20,26,87]. In particular, among patients with SCD, forgetfulness could be exacerbated by undiagnosed or underestimated poor executive functioning and other cognitive deficits experienced in this population [88-90]. Consistent with our findings, earlier studies have reported access barriers and difficulties obtaining refills as a major challenge to hydroxyurea adherence [20,21]. Therefore, understanding patients’ views and perceptions of hydroxyurea and possible access barriers is critical to developing an intervention that is customized to a particular population, increasing its likelihood for adoption and ability to improve adherence levels. We believe *InCharge Health* addresses these important barriers identified in our population and is supported by the literature.

Overall, the results of this study are consistent with prior literature examining barriers to medication adherence and the use of mHealth among diverse and minority populations. For example, in a cohort of urban minority adults with chronic obstructive pulmonary disease, nonadherence to medications was associated with low income, fewer years of informal education, and concerns about medication [91]. These results emphasize the importance of our findings pertaining to suboptimal levels of low health literacy among individuals with SCD and the potential impact on medication adherence and the tailoring of interventions to account for this factor. Within this study, the worry about side effects of hydroxyurea was an identified barrier to adherence, which is consistent with research among low-income, racially diverse adults with type 2 diabetes, which indicated that a common barrier to adherence among these adults was the belief that medications are harmful [92]. Interestingly, forgetting to take the medication was not a common barrier among this sample of adults with type 2 diabetes, unlike this study, where it was the most common barrier reported by patients with SCD. This is perhaps partially indicative of the cognitive effects prominent within SCD [93-95] versus other diseases such as diabetes.

### Mobile Health Solutions to Improve Medication Adherence in Sickle Cell Disease

Recent data reported high access to personal technology, including mobile devices, desktops, laptops, tablets, or iPads, in the general population and among adolescents and adults with SCD [22,41,48,96]. Our study focused on adolescents and adults with SCD who provided invaluable insights into the most suitable platform for technology-based interventions in our population. mHealth interventions represent a promising approach for improving hydroxyurea adherence in patients with SCD, including text messaging or directly observed therapy using mobile phones [59-61,97,98]. In our study, we also evaluated participants’ preferences for a mobile phone app that would help to promote hydroxyurea adherence. Our participants identified medication reminder/tracker, education, communication, personalization, motivation, support during the pain episode, and social support as the most important features for their SCD-specific app. Our findings were consistent with our preliminary data that reported similar mobile phone app preferences among adolescents and young adults with SCD [22]. However, a unique feature of our study, and contribution to this body of work, is our inclusion of both adolescents and adults (aged 18-45 years) and taking a user-centered approach from concept elicitation to mobile phone app development (*InCharge Health*). In addition, our study was informed by an established theoretical model, the HBM [39], at different phases of the project. Recent evidence from several systematic reviews of medication adherence interventions [53-56,58,99,100] and experience in patients with SCD [22,58], cystic fibrosis [101], diabetes [102], and chronic pain [103] suggest that a personalized user-centered approach with a multi-functional mobile phone app may have the potential to improve low hydroxyurea adherence among adults with SCD. This approach is more likely to help overcome adherence barriers and engage participants with the app over time.

Although the use of mHealth among diverse and minority populations has often been an understudied area within the broader digital health intervention literature, this study adds to a quickly evolving field that places increased emphasis on examinations of acceptability and efficacy of mHealth interventions for underserved and minority populations. For example, there have been recent examinations of mHealth interventions (eg, text message, app, and social media based) to increase medication adherence among HIV-positive men who have sex with men [104], of app-based and culturally tailored self-management programs targeting blood pressure among hypertensive Hispanic adults [105], and of a text messaging–based intervention designed to increase medication adherence among low-income, diverse adults with type 2 diabetes [92]. Recent work has also provided evidence for the acceptability of using an app-based assessment of breast cancer risk among ethnically diverse, older, and low-income women [106] and the reliability and validity of mobile phone-based self-monitoring among African American and Latina mothers [107]. Despite this progress, it is still important to note that although the number of individuals with access to the internet and mobile phones continues to rise, varying levels of access and mHealth usage still exist among vulnerable populations nationally and internationally [58,108-111].

### Limitations

Our study had a few limitations that are worth noting. First, data were collected from patients treated at academic institutions and members of a community-based organization, all in Memphis, as a convenience sample of adults with SCD, which might limit the generalizability of our results to other sickle cell centers. Second, selection bias can be a concern where motivated patients are more likely to participate in clinical research studies. However, our interviews were intentional at enrolling patients during their regular clinic visits, potentially reducing a selection of those most motivated to attend a separate research visit. Patients with SCD who were not compliant with their clinic visits or did not participate in the study for different reasons (eg, refusal) may have provided different insights and additional information to inform app development. Despite the potential selection of motivated participants, our results on mobile phone app preferences were consistent with published studies [22,48]. Third, in addition to the NVS, which is a validated tool that was tested in SCD, we used another nonvalidated survey instrument to examine hydroxyurea barriers and facilitators. Nevertheless, our survey was informed by an established behavioral theory (ie, HBM) and developed by experts in the field. Fourth, the wide age range within this study somewhat limits the extent to which the app design and content can be tailored to fit the needs and preferences of individuals with SCD of all ages. Finally, we did not examine patients’ access to different personal technology tools (eg, iPhone or Android mobile phones, tablets, or laptops). However, prior studies reported wide access to these devices among adolescents and adults with SCD [22,47,48], similar to the general population [40,41].

### Conclusions

In conclusion, patients with SCD and health care providers identified several challenges with optimal hydroxyurea adherence. Our findings of barriers to hydroxyurea utilization replicated those in the literature, utilized the HBM as the conceptual framework for understanding the health behavior of hydroxyurea adherence, and directly informed the translation of mHealth into an intervention to modify hydroxyurea adherence. The invaluable insights of patients with SCD on several preferred app features guided, in all stages of the design, the development of a novel adherence-enhancing multicomponent mobile phone app, *InCharge Health*, focused on hydroxyurea adherence. The efficacy and implementation of *InCharge Health* as an mHealth intervention to promote hydroxyurea adherence will be tested in a multicenter trial (NCT04080167) [112]. If this new mHealth intervention is proven efficacious, it can be incorporated into the regular care of patients with SCD as an adjuvant to improve medication adherence. Given the existing evidence, mHealth interventions have considerable potential to optimize medication adherence, quality of life, and health outcomes in patients with SCD and other chronic health conditions.

## Appendix

Multimedia Appendix 1 Phase 2 participant characteristics by age group (N=99).

Multimedia Appendix 2 Optimization digital innovation workshop for hydroxyurea.

## Abbreviations

AHRQ: Agency for Healthcare Research and Quality
FDA: Food and Drug Administration
HBM: Health Belief Model
mHealth: mobile health
NHLBI: National Heart Lung and Blood Institute
NIH: National Institutes of Health
NVS: Newest Vital Sign
SCCRIP: Sickle Cell Clinical Research and Intervention Program
SCD: sickle cell disease
SCDIC: sickle cell disease implementation consortium
