# Supplementary material for: Development of the InCharge Health Mobile App to Improve Adherence to Hydroxyurea in Patients With Sickle Cell Disease: User-Centered Design Approach
Source: JMIR Mhealth Uhealth. 2020 May 8;8(5):e14884. doi: 10.2196/14884 (PMC7245000; doi:10.2196/14884)
Supplement: Multimedia Appendix 1 [file mhealth_v8i5e14884_app1.docx]

**Multimedia Appendix 1: Phase 2 Participant Characteristics by age group (N=99)**

|  | Adolescents  (15-17 years old)  (n=78) | Adults  (18-45 years old)  (n=21) | Total  (n=99) |
| --- | --- | --- | --- |
| **Sex, N (%)** |  |  |  |
| Female | 37 (47.4) | 9 (42.9) | 46 (46.5) |
| Male | 41 (52.6) | 12 (57.1) | 53 (53.5) |
| **SCD Genotype, N (%)** |  |  |  |
| HbSS | 49 (62.8) | 13 (61.9) | 62 (62.6) |
| HbSβ^0^-thalassemia | 7 (9.0) | 1 (4.8) | 8 (8.1) |
| HbSC | 18 (23.1) | 5 (23.8) | 23 (23.2) |
| HbSβ^+^-thalassemia | 4 (5.1) | - | 4 (4.0) |
| Other variant | - | 1 (4.8) | 1 (1.0) |
| Don't know | - | 1 (4.8) | 1 (1.0) |
| **Annual Household Income, N (%)** |  |  |  |
| < $5,000 | 23 (29.5) | - | 23 (23.2) |
| $5,000-19,999 | 8 (10.3) | 6 (28.6) | 14 (14.1) |
| $20-49,999 | 9 (11.5) | 6 (28.6) | 15 (15.2) |
| $50-94,999 | 5 (6.4) | 3 (14.3) | 8 (8.0) |
| ≥ $95,000 | 1 (1.3) | - | 1 (1.0) |
| Prefer not to answer | 10 (12.8) | 3 (14.3) | 13 (13.1) |
| Don't know | 22 (28.2) | 3 (14.3) | 25 (25.3) |
| **Race, N (%)** |  |  |  |
| African-American | 75 (96.2) | 19 (90.5) | 94 (94.9) |
| Other | 3 (3.8) | 2 (9.5) | 5 (5.1) |
| **Ethnicity, N (%)** |  |  |  |
| Non-Hispanic | 78 (100) | 20 (95.2) | 98 (99.0) |
| Hispanic | - | 1 (4.7) | 1 (1.0) |
