# Supplementary material for: Development of the InCharge Health Mobile App to Improve Adherence to Hydroxyurea in Patients With Sickle Cell Disease: User-Centered Design Approach
Source: JMIR Mhealth Uhealth. 2020 May 8;8(5):e14884. doi: 10.2196/14884 (PMC7245000; doi:10.2196/14884)
Supplement: Multimedia Appendix 2 [file mhealth_v8i5e14884_app2.pdf]

Multimedia Appendix 2: Optimization Digital Innovation Workshop for Hydroxyurea

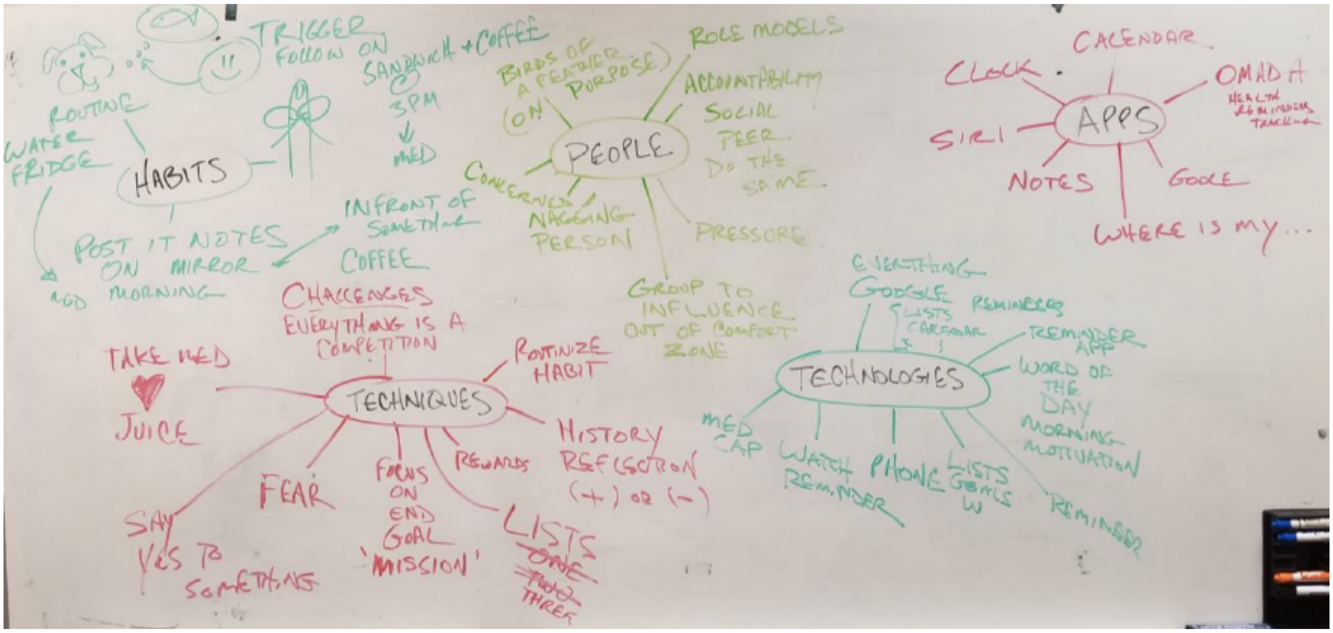

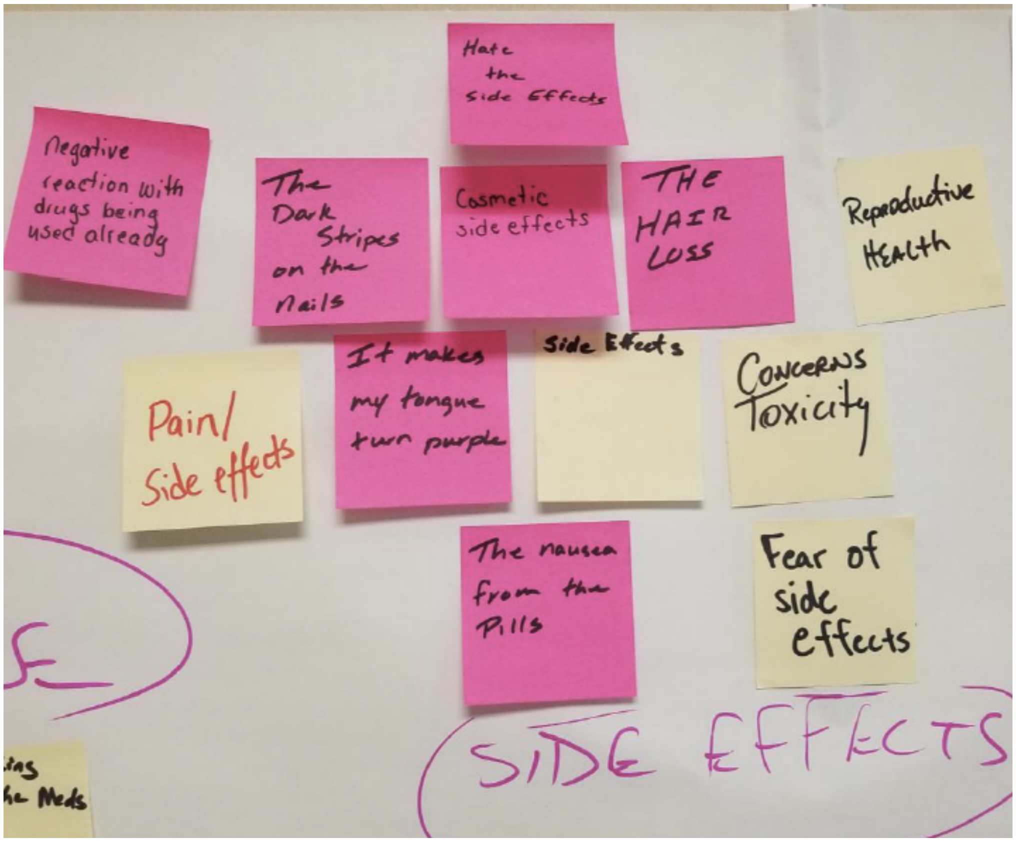

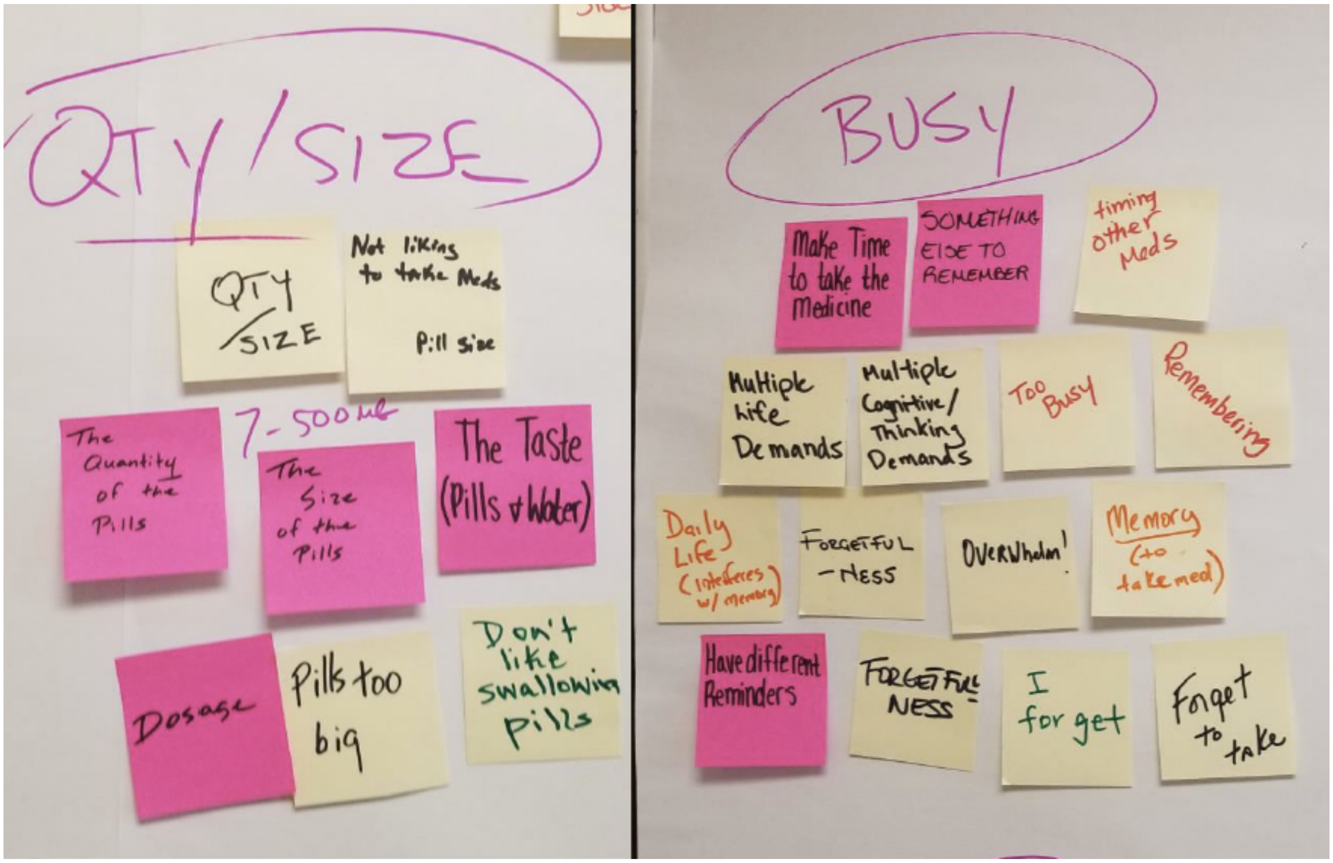
QTY/SIZE
QTY/SIZE
Not liking to take Meds
Pill size
The Quantity of the Pills
7-500 ML
The Size of the Pills
The Taste (Pills & Water)
Dosage
Pills too big
Don't like swallowing pills
BUSY
Make Time to take the Medicine
SOMETHING ELSE TO REMEMBER
timing other Meds
Multiple Life Demands
Multiple Cognitive/ Thinking Demands
Too Busy
Remembering
Daily Life (interferes w/ memory)
FORGETFUL -NESS
OVERWhelm!
Memory (to take med)
Have different Reminders
FORGETFUL NESS
I for get
Forget to take

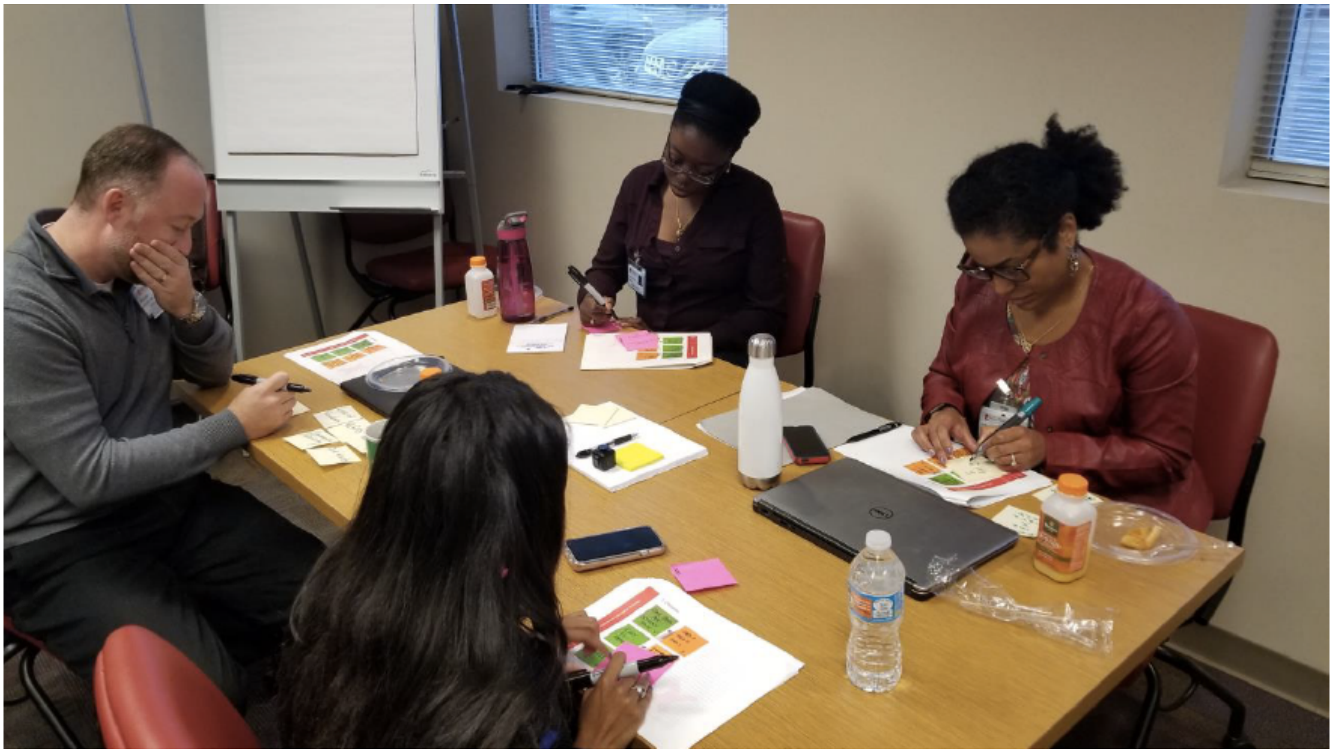

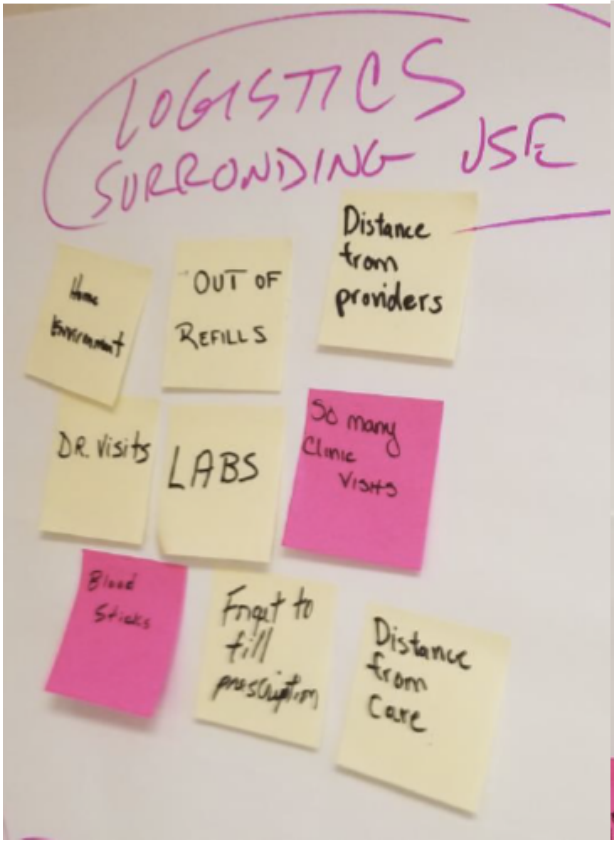
LOGISTICS SURROUNDING USE
Home Environment
OUT OF REFILLS
Distance from providers
DR. Visits
LABS
So many Clinic Visits
Blood Sticks
Forgot to fill prescription
Distance from Care

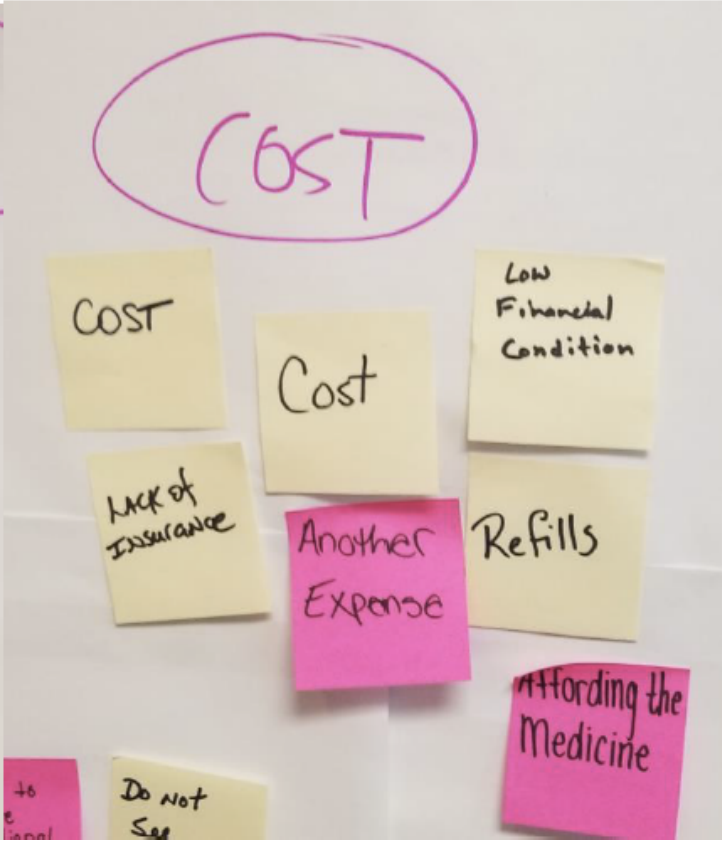
COST
COST
Cost
Low Financial Condition
LACK of INSURANCE
Another Expense
Refills
Affording the Medicine
Do Not

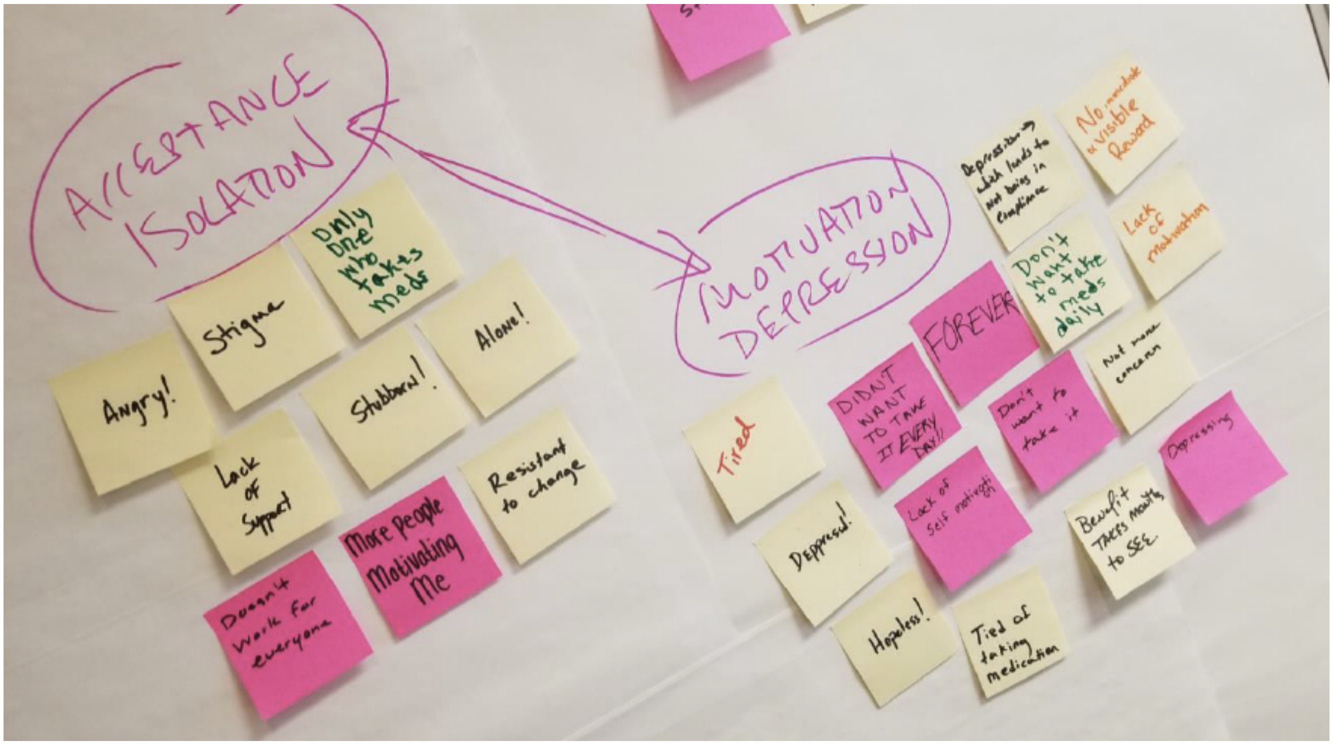
ACCEPTANCE ISOLATION
Only one who takes meds
Stigma
Angry!
Stubborn!
Alone!
Lack of Support
Resistant to change
More people Motivating Me
Doesn't work for everyone
MOTIVATION DEPRESSION
FOREVER
Don't want to take meds daily
Lack of motivation
DIDNT WANT TO TAKE IT EVERY DAY!!
Don't want to take it
Not more concern
Depressing
Tired
Depressed!
Lack of self motivation
Benefit TAKES months to SEE.
Hopeless!
Tired of taking medication

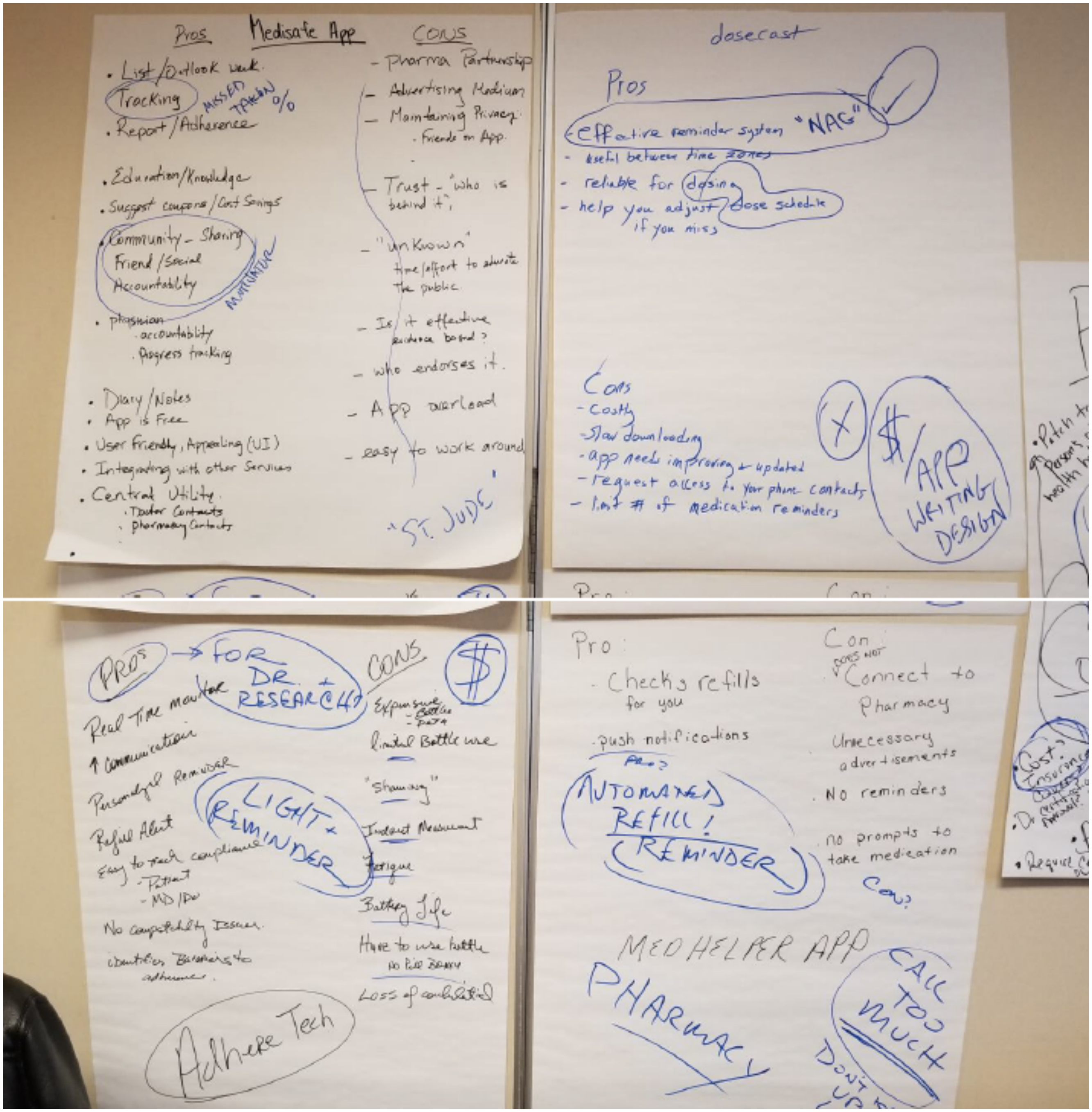
Pros
Medisafe App
CONS
List/Outlook week.
Tracking
Report/Adherence
Education/Knowledge
Suggest coupons/Cost Savings
Community - Sharing
Friend/Social
Accountability
accountability
Progress tracking
Diary/Notes
App is Free
User Friendly, Appealing (UI)
Integrating with other Services
Central Utility.
Doctor Contacts
Pharmacy Contacts
- Pharma Partnership
- Advertising Medium
- Maintaining Privacy.
Friends on App.
Trust - "who is behind it"
"unknown"
time/effort to educate the public
- Is it effective
- who endorses it.
- App overload
- easy to work around
"ST. JUDE"
dosecast
Pros
effective reminder system "NAG"
- useful between time zones
- reliable for dosing
- help you adjust dose schedule if you miss
Cons
- Costly
- Slow downloading
- app needs improving + updated
- request access to your phone contacts
- limit # of medication reminders
$/APP WRITING DESIGN
PROS
FOR DR. + RESEARCH
CONS
Real Time monitor
↑ communication
Personalized Reminder
Refill Alert
Easy to track compliance
- Patient
- MD/Dr
No compatibility Issues.
Limited Bottle use
"Shaming"
Instant Measurement
Fatigue
Battery Life
Have to use bottle
Loss of confidentiality
LIGHT + REMINDER
Adhere Tech
Pro
- Checks refills for you
- push notifications
AUTOMATED REFILL! REMINDER
Con
DOES NOT Connect to Pharmacy
Unnecessary advertisements
. No reminders
. no prompts to take medication
MEDHELPER APP
PHARMACY
CALL TOO MUCH
Cost?
